# Cost-Effectiveness of More Intensive Blood Pressure Treatment in Patients with High Risk of Cardiovascular Disease in Saudi Arabia: A Modelling Study of Meta-Analysis

**DOI:** 10.1155/2019/6019401

**Published:** 2019-09-30

**Authors:** Ziyad Almalki, Yasser Alatawi, Adnan Alharbi, Bader Almaklefi, Suliman Alfaiz, Omar Almohana, Yasser Alsaidan, Abdullah Alanezi

**Affiliations:** ^1^Department of Clinical Pharmacy, College of Pharmacy, Prince Sattam Bin Abdulaziz University, Al-Kharj, Riyadh, Saudi Arabia; ^2^Department of Clinical Pharmacy, College of Pharmacy, Umm Al-Qura University, Mecca, Riyadh, Saudi Arabia

## Abstract

**Objective:**

The current literature suggests that more intensive blood pressure (BP) treatment is clinically more effective than less intensive treatment in patients at high risk for cardiovascular disease (CVD). In this analysis, we evaluated the potential clinical benefit and cost-effectiveness of more intensive BP treatment in patients at high risk of developing CVD over their lifetimes.

**Methods:**

A Markov state-transition model was developed for the BP strategies to estimate the lifetime incremental cost-effectiveness ratio (ICER) per quality-adjusted-life-year (QALY) using evidence published from a meta-analysis. The other model inputs were retrieved from previous studies. Estimated costs were collected from five hospitals in Riyadh. The model used a lifetime framework adopting Saudi payer perspective and applied a 3% annual discount rate. Sensitivity analysis was conducted using one-way and probabilistic sensitivity analysis (PSA) to evaluate the robustness and uncertainty of the estimates.

**Results:**

Treating 10,000 patients with high CVD risk with more intensive BP therapy would avert a total of 873 CV events over their remaining lifetimes as compared with a less intensive strategy. The projections showed that more intensive BP therapy would be cost-effective compared to the less intensive strategy with incremental costs per QALY of $20,358. Probabilistic sensitivity analysis suggested more intensive control would be cost-effective compared with the less intensive control of BP 87.25 % of the time.

**Conclusion:**

The result of this study showed that more intensive BP treatment appears to be a cost-effective choice for patients with a high risk of CVD in Saudi Arabia when compared with a less intensive BP strategy. Thus, this finding provides strong evidence for the adoption of this strategy within the Saudi healthcare system.

## 1. Introduction

Cardiovascular disease (CVD) is recognized as a major cause of morbidity and mortality worldwide. The World Health Organization (WHO) reported that 17.9 million individuals died from CVDs in 2016, accounting for 31% of all-cause mortality globally [1]. Limited evidence exists on the prevalence of CVD-related mortality in Saudi Arabia. However, the WHO reported that 37% of deaths in Saudi Arabia could be attributed to CVDs [2], which is higher than the global estimate. According to the Saudi Heart Association, the economic burden of CVDs in Saudi Arabia will increase from $3.5 billion in 2016 to $9.8 billion by 2035 after taking into account the effects of population aging and growth [3].

Modification of CVD risk factors is essential to reduce morbidity, mortality, and cost of care in patients at high risk of CVDs. The most important modifiable risk factor of CVDs is blood pressure (BP). In Saudi Arabia, nearly 19% of adults have high BP [2], with 56.6% at higher risk of developing CVD [4]. In recent years, there has been an increased focus on intensive BP-lowering treatment in the routine follow-up of people at high risk of CVD. Previous studies affirmed the benefits of more intensive BP treatment in reducing major cardiovascular events (CVEs) and all-cause mortality in patients at high risk [5]. For example, recently, the Systolic Blood Pressure Intervention Trial (SPRINT) compared the effects of more intensive BP treatment with standard treatment among high-risk adults. The trial showed that intensive BP treatment minimized the rates of CVEs by 25% and mortality by 30% among participants [6].

Owing to the economic and health burden of CVDs in Saudi Arabia, healthcare providers and decision-makers must implement an improved strategy to reduce the prevalence and economic burden of CVDs. However, controversy exists regarding the strategy to intensively lower BP in patients at high risk of CVDs. Although the clinical benefit of this strategy has been evaluated by subsequent trials in people with a higher risk of CVD, different targets have yielded inconsistent results [7–23]. A recent meta-analysis integrated all relevant trials and found that achieving a lower BP could have a considerable reduction in the incidence of major CV complications among patients who had either established CVD or additional CV risk factors. For instance, patients who received intensive treatment had lower incidence rates of CVD events (14%), myocardial infarction (13%), and stroke (22%) [24].

While intensive BP control may reduce the risk of CVD in high-risk patients and consequently reduce the healthcare cost associated with the complications, such a strategy also has essential tradeoffs. Patients need additional treatments and physician visits to reach a lower target of BP [25]. Thus, lifetime benefits must be weighed against higher healthcare implementation costs. These data will be valuable for health policymakers and healthcare providers to make informed choices about treatment for people with increased risk for CVD.

To date, no study has assessed the long-term effect and cost-effectiveness of more intensive BP strategy in high-risk patients in Saudi Arabia. Multiple studies in the United States, China, and the United Kingdom modeled the cost-effectiveness of more intensive BP treatment among high-risk patients [26–29]. The results of these studies provide strong evidence of the cost-effectiveness of intensive BP control.

The importance of determining the most cost-effective treatment strategy for this population is to determine how resources can be allocated to give the greatest benefit. Therefore, the main objective of the present study was to assess the clinical benefit and cost-effectiveness of more intensive BP treatment measures in comparison to less intensive BP treatment measures among high-risk patients over 30 years in the Saudi Arabian healthcare setting.

## 2. Methods

### 2.1. Meta-Analysis Study Overview

To evaluate the cost-effectiveness of more intensive BP treatment among high-risk patients in the Kingdom of Saudi Arabia, we used the pooled outcome data that were collected from a recently published meta-analysis of 19 studies. Descriptive information about the study setting, participants, and interventions for each trial included in this analysis is published elsewhere [24]. Briefly, Xie and colleagues previously synthesized data from 19 trials that compared more intensive versus less intensive BP strategies, including 44,989 high-risk patients, with an average age of 63.1 years with 2,496 major CV events and 1,762 all-cause deaths. All the trials were designed as a randomized controlled trial study design with an average of 0–4·9% of lost to follow-up patients (3·8 years mean follow-up). All studies included only patients with an increased risk for CVD (i.e., hypertension and CVD, diabetes, kidney disease, or other CV risk factors) irrespective of different BP targets or different BP changes. The trials were carried out between 1998 and 2015, with ten taking place between 2008 and 2015 [24]. Using a meta-analysis of clinical data in economic studies makes much sense because meta-analysis can provide a more reliable and representative estimate of treatment effects on high-risk patients than a single clinical trial that may include only a specific type of risk [30].

### 2.2. Model Structure

**Figure 1:**
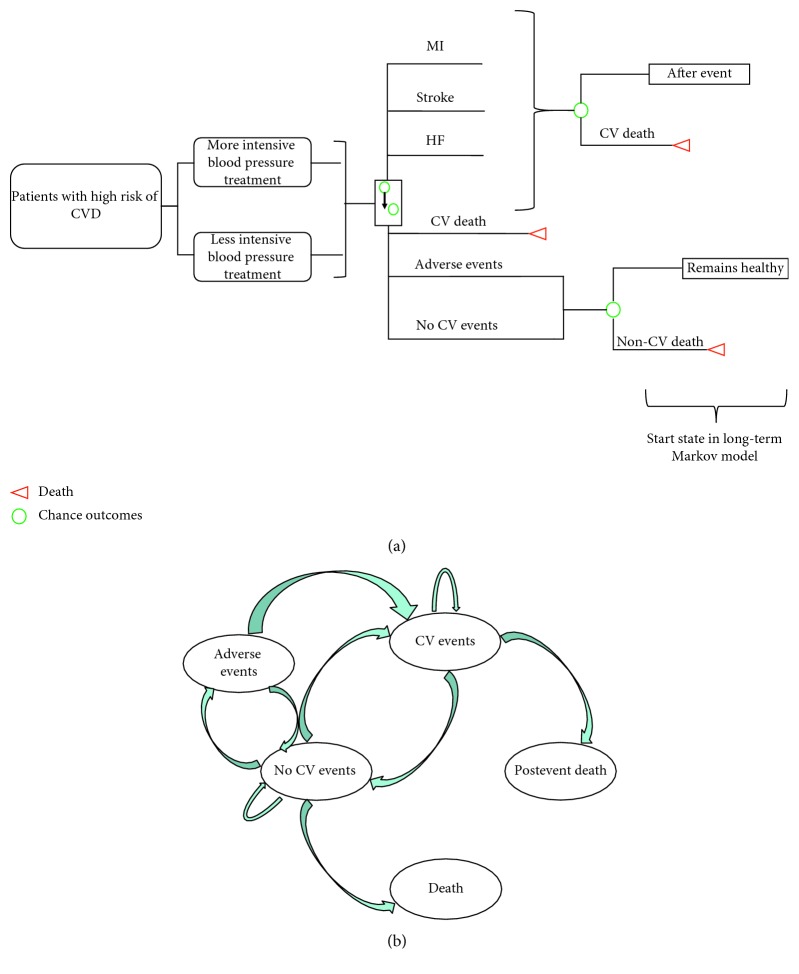
Long-term Markov extrapolation model. (a) Markov model, indicating BP-lowering strategies and possible transitions to CV event states. Abbreviations: MI, myocardial infarction; HF, heart failure; CV, cardiovascular. Green circles represent chance outcomes; red triangles are terminal states. After each cycle, patients either remain healthy or experience a clinical event (MI, stroke, HF, and death, or adverse events). (b) Long-term Markov extrapolation model.

A lifetime Markov process model was constructed to examine health economic benefits, and QALY gained was associated with stroke, myocardial infarction (MI), heart failure (HF), and CV death prevention when more intensive BP treatment was used in comparison with less intensive BP treatment. Our Markov model depends on two hypothetical and identical groups of individuals who are at high risk of CV disease: less intensive BP control and more intensive BP control. Both groups had probabilities of transitioning into 1 of 6 health states each year (i.e., MI, stroke, HF, CV death, no CV event state, or occurrence of adverse events). At the end of every year, the individuals are then redistributed into 1 of 6 health states. When individuals entered a disease state, they either progressed through the health state (i.e., post-CV event state) or died from it (i.e., CV death). The state Deaths from Other Causes (i.e., Non-CV Death) was created because patients may die from other causes not related to CV. The structure of the Markov model is shown in Figure 1. Each of the health states and substates has associated costs and effectiveness. Per the ISPOR Good Research Practices Task Force recommendations [31], the analysis was conducted over a lifetime horizon with a maximum of 30 years. All future costs (in U.S. dollars) and benefits were discounted at 3% annually following the new US Panel on Cost-Effectiveness in Health and Medicine for their recommendations [32]. To reduce the approximation error, the half-cycle correction was applied.

To assess the quality and accuracy of the Markov model, we compared the predicted number of outcome events in the more intensive BP treatment and standard BP treatment groups to those in the meta-analysis data. The model closely predicted the number of outcome events observed in both arms of the meta-analysis during the mean follow-up period of 3.8 years.

### 2.3. Variables in the Model

Probabilities of transition to the next health state, health outcomes, and costs are summarized in Table 1.

### 2.4. Transition Probabilities

The probability of each health state was gathered from the published meta-analysis study and supplemented by other literature. Mortality rates from stroke, MI, and heart failure were estimated from previous literature. Age-dependent non-CV death rates were applied in the model using the age-specific U.S. life table data [43]. To project the risk of CV events beyond the 3.8 years of mean follow-up years of trials included in the meta-analysis, annual transition probabilities for each health state were estimated using standard statistical methods allowing for the length of the cycle [44].

### 2.5. Costs

For this model, we assessed direct medical costs by summing the costs for acute hospitalization, costs of ongoing care for patients with a stroke, physician services, and medication use. We considered the Saudi payer perspective, because currently all Saudi population receives healthcare free of charge without any copays. Due to the scarcity of cost data for clinical events in governmental hospitals, costs for acute CV events, adverse events, stroke rehabilitation cost, and costs of physician visits were collected in August 2018 from five private hospitals in Riyadh, Saudi Arabia. The annual average cost of antihypertensive drugs (per person per year) was calculated using four steps. First, we identified a list of antihypertensive medications used in high-risk patients in Saudi Arabia using data published by Alavudeen et al. [45]. The cost of each medication was then retrieved from the Saudi Food and Drug Authority website [46]. Then, medication costs were calculated by dividing the sum of the lowest wholesale prices for all medications by their total number of prescriptions. Given that patients required, on average, three antihypertensive medications compared with two for those on a less intensive strategy [6], the retrieved average cost of antihypertensive medication was multiplied by 3 and 2 to obtain the daily cost of antihypertensive drugs in more intensive and less intensive strategies, respectively. We assumed that the distribution of antihypertensive drug classes between both groups is similar.

The cost of physician visits included the costs of office visits and laboratory monitoring. We assumed that those in the more intensive BP treatment arm saw their physicians about three times a year and those in the less intensive BP treatment arm were seen twice-yearly [27]. All costs and estimates have been adjusted to 2018 U.S. dollars using exchange rates for currency conversion and have been rounded to the nearest dollar.

### 2.6. Health Outcomes

According to standards set by the U.S. Panel on Cost-Effectiveness in Health and Medicine, quality-adjusted life years (QALYs) were used as a summary measure of health outcome, which considers the number of years lived adjusted to their quality [47]. All QALYs were retrieved from the published literature. Utilities were adjusted by age for each health state in the model using age-specific utility based on Belgian data [48]. Because country-specific utilities were not available in Saudi Arabia, all utilities were varied over a wide range in the sensitivity analysis. Also, we incorporated the disutility for taking more medications for CV prevention.

### 2.7. Analysis

An incremental cost-effectiveness analysis was undertaken. QALYs, costs, and the number of CV events were reported. To test the robustness of our results, we performed one-way sensitivity analyses to different variables, including the probabilities of various disease states, costs, and utilities to increase the generalizability of this model. In these analyses, we modified each input parameter value to determine the individual impact on the results by using 95% confidence intervals (CI) if available or by varying costs by 50% and by 20% for other parameters. The results of the one-way sensitivity analysis are then illustrated in the tornado diagram. Probabilistic sensitivity analyses (also known as second-order Monte Carlo simulation) were also conducted by a varying number of model inputs to assess the joint uncertainty of model inputs on the overall results. In this analysis, gamma distributions were used to model costs and beta distributions were used to model the probability and utility values. A cost-effectiveness acceptability curve (CEAC) was constructed to depict the probability of a strategy being cost-effective at different cost per QALY willingness-to-pay (WTP) thresholds. Given that no threshold is defined by Saudi Health Authorities, we determined the WTP threshold using the most cited approach in global health in recent years, which has been recommended by the World Health Organization (WHO) Choosing Interventions that are Cost-Effective (CHOICE) program. The WHO recommended choosing interventions that cost less than three times the national annual gross domestic product per capita (GDP/capita). This figure would be approximately $60,000/QALY as the GDP figure is $20,796/capita from 2017 [49]. All analyses were performed in TreeAge Pro 2019 software (TreeAge Software, Inc, Williamstown, MA, USA).

## 3. Results

The number and type of events for 10,000 patients 63 years of age at high risk of CVD in both treatment groups estimated by using the Markov model are presented in Table 2. Treating high-risk patients with a more intensive BP strategy over 30 years would yield a reduction of approximately 142 CV deaths, 341 strokes, 241 MI events, and 149 HF events. The mean number of QALYs would be 0.58 higher among people who received more intensive BP treatment than those who received less intensive BP treatment and would cost approximately $20,358 per QALY gained.

**Figure 2:**
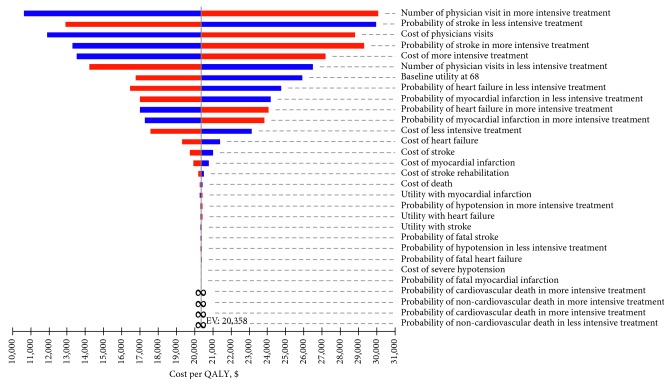
Tornado diagram of multiple one-way sensitivity analyses.

One-way sensitivity results are shown in the tornado diagram in Figure 2, which shows how the variations in each model input affect the overall results. As shown in the figure, the bars are arranged in order in decreasing width, with the widest bar at the top, indicating that variations in inputs (number of physician visits in more intensive treatment, probability of stroke in less intensive treatment, cost of physician visit, probability of stroke in more intensive treatment, cost of intensive treatment, and number of physician visit in less intensive treatment) have the most significant effect on the outcome, while the narrowest bar at the bottom indicates that variations in inputs (probability of MI, probability of CV death in more intensive treatment, probability of non-CV death in more intensive treatment, probability of CV death in more intensive treatment, and non-CV death in less intensive treatment) have relatively small effects on the outcome.

**Figure 3:**
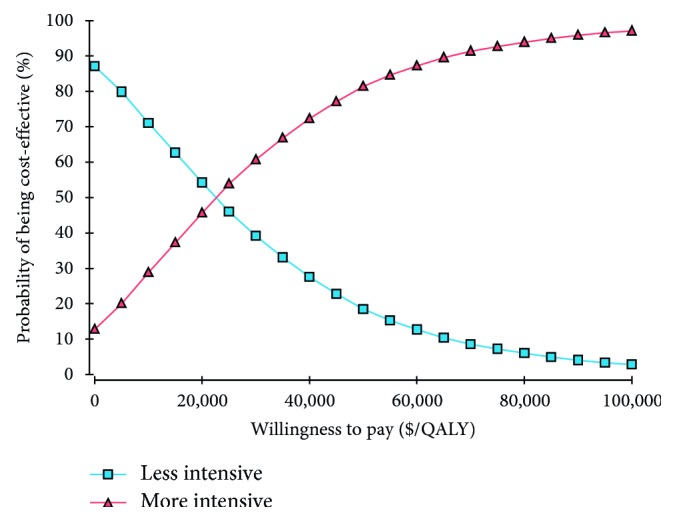
Cost-effectiveness acceptability curve.

The results of probabilistic sensitivity analysis, using a Monte Carlo simulation of 10,000 samples, at given different values of willingness to pay are shown in Figure 3. Compared with less intensive treatment, the more intensive one was cost-effective in 87.25% of simulations at a threshold level of $60,000/QALY.

## 4. Discussion

Intensification of antihypertensive therapy has been controversial in high-risk patients [50]. To adopt this strategy in practice, it was essential to consider the balance between benefits and harms in individual patients using an evidence-based synthesis of new accumulating data that include all types of high-risk populations so these data would be readily generalizable to high-risk patients. According to evidence derived from a recent meta-analysis aimed to quantify the benefits and harm of the more intensive BP treatment, more intensive BP treatment provided greater vascular protection than less intensive BP treatment [28].

In this study, we developed a Markov model to project the cost-effectiveness of the more intensive BP treatment strategy among adults at high risk for cardiovascular disease using data from the only meta-analysis published that focused on high-risk patients. From the perspective of Saudi payer, we found that more intensive BP treatment was cost-effective over the course of a lifetime. The baseline Markov model was sensitive to changes that were made in costs. The results show that this approach becomes even more favorable if the cost of treatment and monitoring could be reduced.

It appears that the costs associated with implementing more intensive treatment are balanced by the thousands of CV events and subsequent treatment costs that were prevented. According to our findings, more intensive treatment could avert a substantial number of CV events in the next 30 years for patients at high risk of CVDs in Saudi Arabia.

Our findings are in agreement with previous cost-effectiveness studies of more intensive treatment, which have found that managing hypertension using more intensive BP treatment is cost-effective or even cost-saving. For instance, a cost-effectiveness analysis study that evaluated the cost-effectiveness of an intensive treatment lowering BP levels compared with a standard treatment in a high-risk population over a 30-year time horizon found that the more intensive BP treatment was extremely cost-effective [28]. Another cost-effectiveness study found that the more intensive BP treatment in high-risk patients in England was a cost-effective strategy, with a cost per QALY of £6,927 [51].

In this study, the time horizon of 30 years was selected because, first, patients in Saudi Arabia receive full and free coverage from the Ministry of Health (MOH), including medications without any copays, and rarely individuals discontinue MOH coverage to buy insurance plans [52]. The second reason for selecting the time horizon of 30 years was to account for the long-term nature of the disease since the majority of patients would be expected to have died within that period of time, given the mean age range at baseline in the meta-analysis study (i.e., 63 years) [24].

Our results are important not just because they show that the more intensive BP treatment is cost-effective but because of their population-wide implications. According to the previous report, 56.6% of hypertensive adults are at high risk of CVD and stand to benefit from more intensive BP treatment [4, 53]. We recommend the implementation of more intensive BP treatment for patients who are at high risk for CVD with close monitoring for serious adverse events in practice. The same action has been taken by Canada and Australia [54, 55].

Our study had several limitations. First, we did not include indirect costs of patient care, such as transportation and caregiver costs. The second limitation of the study is the lack of published cost studies that are specific to the Saudi Arabian populations, which is a significant problem throughout the developing world [56]. Third, because this study was based on a meta-analysis data that included high-risk patients, with an average age of 63 years, the results may not generalize to younger population or/and with a lower risk of CVD. A related limitation is that the effects of clinical treatment data were retrieved from a pooled estimate of trials conducted in other countries. Also, using the Belgian utility values and the absence of the local value sets carries some risks in not representing the views and preferences of the Saudi population. We, therefore, performed sensitivity analyses on these input data which showed that the variations in these parameters would not appear to be an issue in this instance. Finally, the BP target in the treatment groups differed across the individual trials involved in the meta-analysis study. As such, we are not able to evaluate the cost-effectiveness of more versus less intensive BP treatment at different BP targets, so we could not determine the optimal target of BP control economically.

To conclude, with an increased prevalence of CVD in Saudi Arabia and life expectancy, the rates of CV complications would be much higher, which would place more substantial demand on Saudi healthcare expenditure. Thus, policymakers need information not only on the clinical effectiveness of an intervention but also on whether it provides economic value concerning the cost of implementation of that intervention. In our study, we used clinical data from the meta-analysis study, the findings of which indicate that more intensive BP treatment in patients at high CV risk is a highly cost-effective intervention. The findings are robust, being reproduced across several sensitivity analyses. Future studies should incorporate indirect costs to allow for a full economic evaluation of both BP treatment strategies.

## Figures and Tables

**Table 1:** The inputs of the economic model.

Variable	Value	Range used in sensitivity analyses^b^	Distribution used in probabilistic sensitivity analyses	Reference no.
*Event rates (per year)*				
*More intensive blood-pressure control*				
Risk of CV outcomes				
MI	0.0048	0.0038 to 0.0057	Beta	[24]
Stroke	0.0059	0.0047 to 0.0071	Beta	[24]
Heart failure	0.0028	0.0022 to 0.0034	Beta	[24]
CV death	0.0046	0.0039 to 0.0055	Beta	[24]
Non-CV death	0.0049	0.0039 to 0.0058	Beta	[24]
Risk of adverse event, hypotension	0.0024	0.0019 to 0.0028	Beta	

*Less intensive blood-pressure control*				
Risk of CV outcomes				
MI	0.0054	0.0043 to 0.0065	Beta	[24]
Stroke	0.0065	0.0052 to 0.0078	Beta	[24]
HF	0.0034	0.0027 to 0.0041	Beta	[24]
CV death	0.0048	0.0038 to 0.0057	Beta	[24]
Non-CV death	0.0053	0.0042 to 0.0064	Beta	[24]
Risk of adverse event, hypotension	0.0011	0.0009 to 0.0013	Beta	

*Mortality*				
Fatal MI	0.002	0.0016 to 0.0024	Beta	[30]
Fatal stroke	0.022	0.017 to 0.026	Beta	[33]
Fatal HF	0.009	0.007 to 0.01	Beta	[34]

*Costs ($; year 2018 values)*				
*The average cost of hypertensive drugs (per person per year)*				
More intensive	753	376 to 1,129	Gamma	Estimate
Less intensive	484	242 to 726	Gamma	Estimate

*Costs of acute disease*				
MI	16,720	8,360 to 25,080	Gamma	[35]
Stroke	29,576	14,788 to 44,364	Gamma	Estimate
HF	34,263	17,131 to 51394	Gamma	Estimate
Acute stroke rehabilitation cost (year)	14,627	7,313 to 21,940	Gamma	Estimate
Death	6,000	3,000 to 9,000		

*Cost of adverse event*				
Sever hypotension	1600	800 to 2,400	Gamma	Estimate

*Physician visits*				
Cost of a physician visit	537	268 to 805	Gamma	Estimate
Number of physician visits, *n*				
More intensive	3	2 to 4	Gamma	
Less intensive	2	1 to 3	Gamma	

*State utilities*				
Baseline utility at 63 (high risk individual free of CV or adverse events complications) (per y, unless noted)	0.79	0.63 to 0.95	Beta	[36]
MI^a^	0.70	0.56 to 0.84	Beta	[37, 38]
Stroke^a^	0.57	0.46 to 0.68	Beta	[37, 39]
HF^a^	0.43	0.34 to 0.52	Beta	[37, 40]
Disutility of adverse event, severe hypotension	−0.06	−0.048 to −0.072		[41]
Disutility for taking more medications	−0.002	−0.001 to −0.003	Beta	[42]

^a^These figures are multiplied by initial health state utility to estimate new health state utility. ^b^Sensitivity ranges are based on 95% confidence intervals when available or represent +/50% for costs and +/20% for other parameters. Abbreviations: MI, myocardial infarction; HF, heart failure, CV, cardiovascular.

**Table 2:** Projected health outcomes, costs, and QALY of more intensive vs. less intensive BP strategy.

Outcomes	Less intensive strategy	More intensive strategy	Incremental
*Clinical outcomes in 30 years (per 10,000 patients)*			
Total number of CV deaths	1,333	1,191	−142
Total number of strokes	1,968	1,227	−341
Total number of MI	1,485	1,244	−241
Total number of HF	873	724	−149
Total	5,559	4,686	−873

*Health effects in 30 years (QALYs)*			
Total QALYs	103,644	109,408	5,764
Total QALYs (undiscounted)	127,939	136,248	8,309
Mean QALYs	10.36	10.94	0.58
*Costs ($)*			
Total costs	481,862,925	599,205,821	117,342,896
Total costs (undiscounted)	590,522,950	743,973,695	153,450,745
Mean cost (per patient per year)	48,186	59,920	11,734

*Incremental cost-effectiveness results in 30 years ($/QALY gained)*			
ICER	—	—	20,358
ICER (undiscounted)	—	—	18,467
ICER in 5 years	—	—	44,562
ICER in 10 years	—	—	30,111
ICER in 20 years	—	—	22,425

## Data Availability

The data used to support the findings of this study are included within the article.
